# Innovation and adaptation: The rise of a fentanyl smoking culture in San Francisco

**DOI:** 10.1371/journal.pone.0303403

**Published:** 2024-05-22

**Authors:** Daniel Ciccarone, Nicole Holm, Jeff Ondocsin, Allison Schlosser, Jason Fessel, Amanda Cowan, Sarah G. Mars

**Affiliations:** 1 Department of Family and Community Medicine, University of California, San Francisco, San Francisco, CA, United States of America; 2 Sociology and Anthropology Department, University of Nebraska Omaha, Omaha, NE, United States of America; 3 Community Health Project Los Angeles, Los Angeles, CA, United States of America; University of Connecticut Health Center: UConn Health, UNITED STATES

## Abstract

**Background:**

Illicitly manufactured fentanyls and stimulants are implicated in the escalating US mortality from drug overdose. San Francisco, California (SF) has seen declining fentanyl injection while smoking has increased. Beliefs and behaviors surrounding this development are not well understood.

**Methods:**

The study used rapid ethnography to explore fentanyl and methamphetamine use in SF. The team conducted semi-structured interviews (n = 34) with participants recruited from syringe service programs. Video-recorded smoking sequences (n = 12), photography and daily field notes supplemented interview data.

**Results:**

Difficulty injecting and fear of overdose motivated transitions from injecting to smoking. Fentanyl was extremely cheap—$10/gram—with variability in quality. Foil was the most commonly used smoking material but glass bubbles, bongs and dabbing devices were also popular. No reliable visible methods for determining fentanyl quality existed, however, participants could gauge potency upon inhalation, and developed techniques to regulate dosage. Several participants reported at least hourly use, some reporting one or more grams of daily fentanyl consumption. Smoking was also very social, with people sharing equipment, drugs and information. Participants raised concerns about hygiene and overdose risk to others arising from shared equipment. Reportedly potent fentanyl ‘residue’ accumulated on smoking materials and was commonly shared/traded/stolen or consumed accidentally with diverse preferences for its use.

**Conclusion:**

Our data highlight fentanyl residue as a new overdose risk with potential mismatch between the potency of the residual drug and the recipient’s tolerance. Further, large doses of fentanyl are being consumed (estimated at approximately 50 mg of pure fentanyl/day). Smoking fentanyl has potential health benefits over injecting and may be protective against overdose, but substantial uncertainty exists. However, SF overdose mortality hit a record high in 2023. Recommendations to reduce fentanyl smoking overdose risks through pacing, greater awareness of dosages consumed and checking tolerance of residue recipients are potentially viable interventions deserving further exploration.

## Introduction

There have been significant changes and innovations in the ways in which fentanyl is consumed by people who use opioids (PWUO) in the United States (US), specifically a widespread shift to fentanyl smoking on the West Coast, beginning at least by 2018 [1, 2]. The early portion of the US’s current rise of illicitly manufactured fentanyl (IMF) was largely limited to the Northeast and Midwest Regions, with some increases in the South [3, 4], but by 2018, the West was experiencing the highest relative changes in overdose rate due to its presence [5]. Geographic differences in drug distribution are well documented in the US, with longstanding divides demarcated by the Mississippi River and historical domination by specific drug trafficking organizations, with little response to consumer demand [6]. These divisions are influenced by both high-level drug trafficking and local market-based factors that may play a role in determining the level of fentanyl penetration of the US heroin supply [7, 8]. Fentanyl dominance of the US opioid market may also be seen in the growing variety of forms and products in which fentanyl is found, including in many counterfeit prescription medications [9].

Overdose deaths related to IMF have made up an increasingly large percentage of overdose mortality in the US since 2013 [3, 10]. This constitutes the third wave of overdose deaths, building on rising prescription opioid overdoses in the early 2000s and a marked increase in heroin overdose deaths from 2007 [11]. More recently, combinations of IMF and stimulant-type drugs such as cocaine and methamphetamine have characterized a fourth wave of overdose deaths [12]. Recently, this has also extended to a shift from a predominant US culture of opioid injection to fentanyl smoking in cities like San Francisco, California [1].

A brief history of opiate smoking is warranted to understand better modern trends and transitions in substance use. Although opium had been available in China since approximately the 8^th^ century, its smoking has only been practiced since the 17^th^ century—linked to the spread in previous centuries of the practice of tobacco smoking [13]—and accelerated across social classes through the British opium trade in the first half of the 19^th^ century [13, 14]. In the US, smoking opium was initially restricted to Chinatown settlements in the West until 1870, when a new class of ‘underworld’ white opium smokers began to emerge, engaging in what Courtwright describes as a “social vice, a way of relaxing and indulging with friends” [14]. The argot of the time emphasized this social nature, as well as extolling those individuals who excelled at the practice of smoking, traits which continue into the 20^th^ and 21^st^ centuries among people who use drugs [14]. While early US efforts to curtail opium smoking were largely unsuccessful, later federal legislation, especially the 1909 ban on opium importation for nonmedical uses, i.e. smoking, succeeded in making the practice too risky and expensive to continue, compounded by the diminution of the Chinese population in the US through the 1882 Exclusion Act and anti-Chinese racism more generally [14]. These smokers were instead to switch to morphine and heroin injection, and heroin smoking would remain almost entirely unseen in the US until experimentation by American soldiers in Vietnam in the early 1970s [14].

Heroin smoking originated in China during the first decades of the 20^th^ century, sold as ‘red pills’ that could be smoked in similar fashion to opium [13], although it is thought that heroin was made only marginally bioavailable through this method [15]. These pills held little heroin, and often contained far greater quantities of other substances, including caffeine, quinine, cocaine and colored dyes, and their use is not thought to have extended much past the 1930s [13, 15]. The first form of ‘chasing the dragon’ emerged in Hong Kong during the 1950s, where powdered heroin was combined with barbiturates on foil and heated, allowing for inhalation of the combined vapors [15, 16]. This method of consuming heroin, spread to other parts of Asia and eventually Europe by the 1980s, where it initially co-existed with a culture of heroin snorting and injection but later overtook them as the most common route of administration [15]. In the early 1990s, Des Jarlais et al remarked, ‘no subculture of heroin smoking has ever developed in the United States’ [17], and this has largely remained true until recently [18]; treatment data show only 4 percent reported smoking as their preferred route of heroin use, compared with 70 percent reporting injecting [19]. However, with the emergence of fentanyl as the dominant opioid in the US illicit market and its higher potency relative to heroin, innovation and adaptations in its use, particularly in markets where fentanyl is sold as-is, are of considerable interest [12].

Research into transitions between modes of administration has largely focused on trajectories towards injection use, initially driven by concerns about HIV outbreaks among this population [20], although studies also identified ‘reverse transitions’ away from injection among some PWUO [20, 21]. Historically it has been common to categorize PWUO based on consumption methods or drug form (smokers versus injectors) and to differentiate between licit and illicit use [14]. This practice continues and is often reproduced or repurposed by PWUO themselves (e.g. ‘dopefiends’ [22], ‘sniffers’, ‘chasers’ or ‘injectors’) [20].

Drug source-form plays a role in mode of use, with specific heroin forms identified as preferential for injection (i.e. powdered heroin hydrochloride salt, the form most common in the US) or smoking routes (i.e. base heroin, the form most common in Europe) [6, 18, 20, 23]. Bioavailability of various source-forms may be notably different when consumed through different administration routes, with hydrochloride salts particularly poor at providing measurable effects when smoked [23]. In contrast, fentanyl salts remain stable at temperatures up to 350°C, thus volatilization may be a particularly effective method for fentanyl ingestion [24].

In the context of North American fentanyl use, there has been little research about large-scale transitions in fentanyl mode of use and health outcomes from such transitions. Some PWUO employ techniques including smoking, snorting (aka ‘tooting’) or tasting drugs to determine potency while injection remained their primary route of administration [25]. Research in Canada has found that some preferred smoking fentanyl to injecting because the high lasted longer and for physiological reasons, including loss of venous access [26], while others believed that smoking reduced risk of overdose relative to injecting [27]. Recently, an observational cohort study in San Francisco, California found that from 2019 to 2020 past 30-days injections had decreased precipitously, while days smoking fentanyl increased [1]. The novel uptake of fentanyl smoking in San Francisco and the US West Coast requires further study and could presage a much wider uptake of fentanyl smoking across the US.

## Methods

This study utilized rapid ethnography [28] to explore experiences and trends surrounding fentanyl and methamphetamine smoking in San Francisco, California. The research team, some of whom were meeting in person for the first time, built rapport with one another while refining the interview guide and conducting background observations in June 2022 (no participant recruitment), and returned for one-week each in September and November 2022, to recruit participants (recruitment period: 9/26/2022–11/18/2022). The team conducted semi-structured interviews capturing information regarding substance use progression and preferences, evolutions in mode of use, changes in the local drug supply, experiences of overdose, and other related topics. Interviews (n = 34) were audio-recorded, averaged 60 minutes, and participants were compensated $25. The team also captured videographic sequences of drug consumption (n = 12)—for which participants were compensated an additional $25—and still photographic evidence of various drug samples, substance use equipment, and the built environment. Daily field notes written by the team supplemented the interview and visual data, highlighting key ethnographic observations and themes for further study. The names of all quoted participants have been changed to protect their privacy. Human subjects approval for the study protocol was given by the University of California, San Francisco Human Research Protection Program (IRB), and a Federal Certificate of Confidentiality protects the data.

### Recruitment and sample

Recruitment occurred from three different syringe service programs, one of which, the *Tenderloin Center*, also operated a supervised consumption space in the vicinity of the Tenderloin and South of Market neighborhoods. The Tenderloin is a neighborhood in downtown San Francisco that has been viewed as a ‘containment zone’ for homelessness, substance use and crime [29], making it the priority for several public health initiatives like the *Tenderloin Center*. Participants were aged 18 or older, and self-reported fentanyl and/or methamphetamine use by any route. Program staff introduced potential participants to the research team, who then discussed the study and offered a chance to participate. The informed consent documented was read through by each participant, and consent was obtained verbally to protect participant confidentiality. Exclusion criteria covered those unable to consent due to intoxication or other causes. Participants routinely showed the research team any smoking or injecting equipment and often consented to documentation via photography, with some offering to participate in video-recorded consumption sequences.

### Analysis

Audio recordings were professionally transcribed and checked by the authors for accuracy. The authors drafted analytic memos for each transcript according to the methods of Christopoulos [30] and developed thematic memos to encapsulate themes and ideas generated across multiple interviews. The authors met weekly to discuss emergent themes from the interview transcripts, with ethnographic observations and field notes providing additional context. Photographs were categorized by their contents, and members of the research team transcribed and produced analytic memos for each video-recorded consumption sequence.

## Results

Participants ranged in age from 18 to 53 years, with 31 (91.2%) reporting current smoking practices, while only 18 (52.9%) reported currently injecting despite 30 (88.2%) having a history of injecting and 21 (61.8%) having injected fentanyl. Further demographic and substance use data are available in Table 1. Additionally, the following shorthand is used throughout to indicate substances by mode of consumption for each participant: injects methamphetamine (IM); injects fentanyl (IF); smokes methamphetamine (SM); smokes fentanyl (SF).

**Table 1:** Interviewee demographic data.

**Gender ^a^**			**N (n = 34)**
	Male		17
	Female		11
	Transgender or Nonbinary		3
	Declined or unclear		3
**Age (*years)***			
	18–25		2
	26–33		13
	34–41		3
	42–49		12
	50–57		4
**Race / Ethnicity ^b^**			
	White		24
	American Indian/Alaska Native		5
	Black/African American		3
	Hispanic/Latino		3
	Declined or unclear		3
**Modes of Use & Substances Consumed**			
	Any history of injection		30
		Ever injected fentanyl	21
	Currently injects		18
		Injects methamphetamine (IM)	17
		Injects fentanyl (IF)	15
	Currently smokes		31
		Smokes methamphetamine (SM)	27
		Smokes fentanyl (SF)	28

^a^ Participants were asked: “What gender do you identify as?” Open-ended responses produced some unclear results that were grouped with those who declined to answer.

^b^ Participants were asked: “What ethnicity do you identify as?” Self-reported answers produced the following categories and ability to list multiple resulted in numbers that add to greater than 34.

Through open-ended inquiry, participants shared how an evolving drug supply influenced their substance use behaviors and trajectories, with fear of overdose and vein damage providing significant motivation to transition away from injecting and towards smoking as a dominant mode of use. Participants drew on prior experience smoking other substances, but the outsize overdose risk of fentanyl compared to previously smoked substances required the generation of new knowledge and techniques for regulating potency. Smoking proved to be more social in nature relative to injecting, causing participants to reflect on the changing risk environment for people with varying opioid tolerances and strategies for protecting others. Each of these themes are explored in further detail.

### Responses to changing markets, bodies and overdose risk

#### Changing market

In San Francisco, participants commented that fentanyl arrived as an unadvertised adulterant to heroin but has since been marketed explicitly as fentanyl, or ‘fetty’ as it was often called. Many participants had experience using heroin and commented on the market shift to fentanyl, making heroin difficult or impossible to find. Upon arrival in northern California, Tera, a woman in her 50s (SM; SF) could not find heroin anywhere, saying “that’s how I ended up going to fetty”. Over time, a booming local fentanyl market developed with a wide range of products of varying quality, and consistently cheap pricing—ranging from $5-20/gram but most often quoted at $10/gram. Clay, a man in his 30s (SM; SF), pointed out the range of quality of this low-priced product and also its potential for extreme potency:

*Interviewer: Okay. So, when you do smoke fentanyl, how much are you doing in a session?*

*Clay: It depends on the quality of it, for sure. Anywhere from a point [0.1g] to a quarter-gram or sometimes more if it’s not that good or whatever. I’ve sat down and smoked a gram before in a couple hours, but it just wasn’t very strong at all, you know what I mean? You just burn through it, and it’s like, “Wow, it’s just not making a difference. It’s not doing anything.” […] If it’s really good, you’ll fucking smoke like a hit or two and you’ll be almost falling asleep…*

The low prices coupled with this market volatility may have created a desire for a consistently stronger product which people called ‘clean’ and priced at $20-50/gram. Jamie, a woman in her 30s (SM; SF; IM; IF), attributed the arrival of ‘clean’ to demand for higher quality fentanyl amidst a fall in fentanyl quality and price.

*And I think, like, the quality of, like, the fentanyl itself went way down. I think there was just, like, too much and not enough people buying it anymore, and all the sudden it was, like, cheap, cheap, cheap, and crappy. You know, it all the sudden was, like, $10 a gram or around or less […] I think there was still a demand for, like, higher quality stuff, even if it meant paying more, paying $20, paying $30, $40, $50 a gram.*

**Fig 1:**
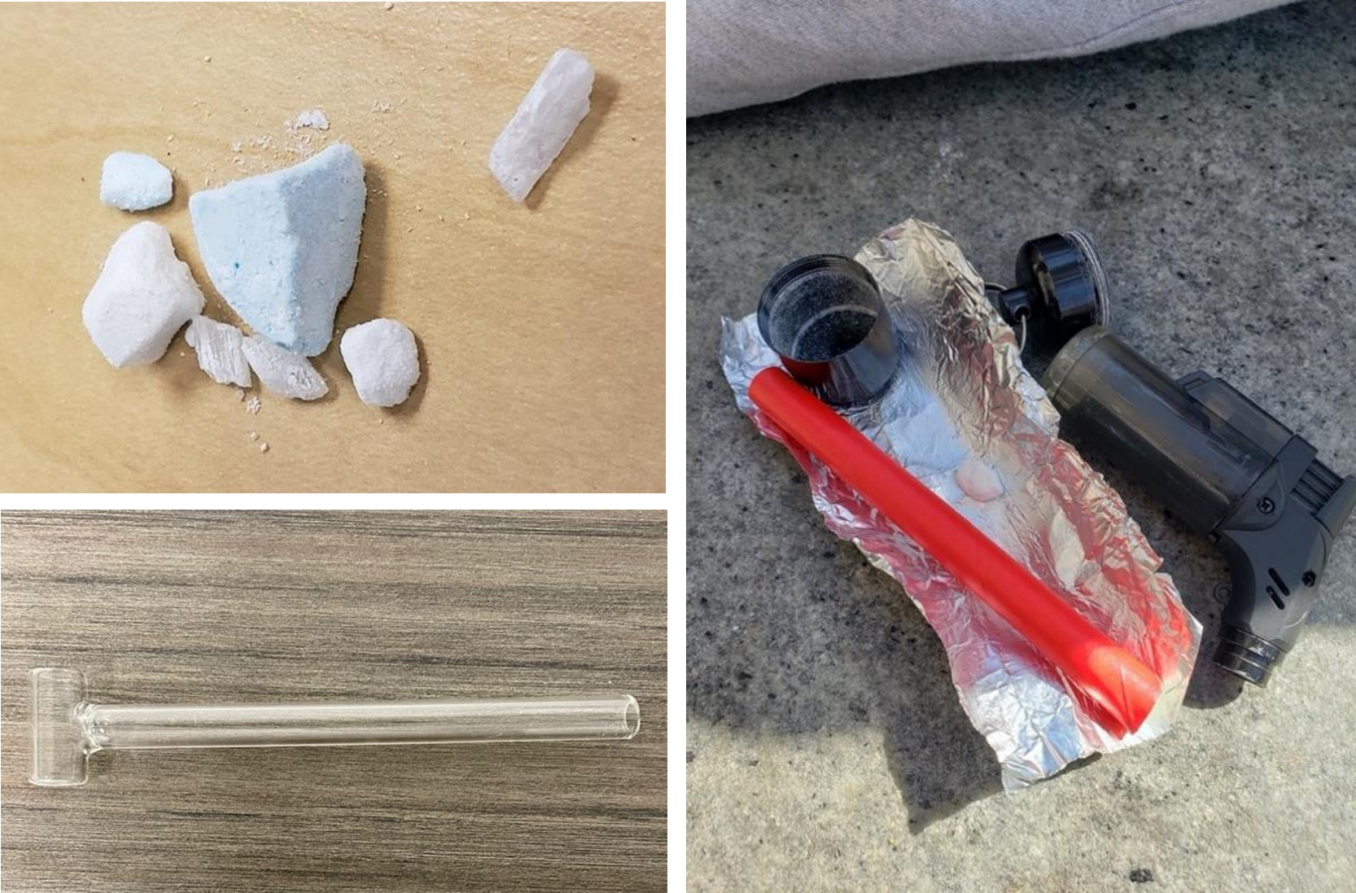
Fentanyl, typically sold in compressed powder chunks, (and a few methamphetamine shards) and the materials commonly used to consume them via inhalation.

In addition to potency, products also ranged in color, texture, and taste (Fig 1) but those cues failed to provide consistent insight into the strength of any given product, as Chris, a man in his 30s (SM; SF), explained:

*Chris: […] The color is not an indicator. The taste is not an indicator. The size, the shape, the–no. Yeah. Or, no, but, like, if you chemically test it, and you know, yeah, this is actually–which, you know, I’d buy a gram of fentanyl. There’s not a gram of fentanyl. There’s probably, like, 20 micrograms of fentanyl in there, not literally but, you know, it’s not a gram of fentanyl.*

*Interviewer: Got you.*

Chris: You know, there’s no way on the streets to determine any of that.

#### Changing bodies

Having previously used heroin, many participants were able to compare experiences and often shared that fentanyl produced a sufficient high by smoking which was not possible with heroin. Josh, a man in his 40s (SM; unclear fentanyl use), shared an experience of accidentally smoking fentanyl and contrasted it against his experience smoking and injecting opium:

*[Smoking fentanyl] felt like pretty much the same as if you were to, like, like, shoot it up or whatever […] it didn’t really, like, how other kinds of heroin, when you smoke, like, other–like, if you smoke opium or things like that, smoking opium feels like smoking opium. It doesn’t feel like–like, you know, shooting up; you know what I mean? Whereas, like, yeah, like, smoking fentanyl, it feels like you, like, shot up a gang of fucking heroin; you know what I mean?*

For several people, this market transition to fentanyl coincided with experiences of injection difficulty due to accumulated vein damage. Russell, a man in his 20s (SM; SF), recounted being completely unable to find accessible veins for heroin injection and welcomed the transition to a more smokable substance.

*Interviewer 2: And what was that, like, transition point from injecting heroin to smoking fentanyl? What was that like?*

*Russell: It was, like, I–I couldn’t get my shots of heroin anymore, so I was just, like, really dope sick, and someone just, like, oh, I have–I have some fentanyl, and I was, like, oh, I don’t want to do that; you know? Stuff–stuff will kill you.*

*Interviewer 1: Yeah.*

*Russell: And, so, finally I was just, like, oh, here, I’ll try it. And I took one hit, and I fell in love. Like, it was like a high; you know? And I hadn’t been high in, you know, probably like a couple months.*

Even for those with high opioid tolerances, fentanyl smoking proved sufficient. Having used heroin intravenously for an extended period, Lana, a woman in her 40s (SM; SF), developed severe difficulty injecting. For her, the discovery of fentanyl smoking was a game changer because its effect was commensurate with injecting. Here she is discussing how this impacted one of her friends in a positive way:

*[…] she would stay in the bathroom hours trying to hit because you can’t find your fucking veins, you know what I mean? It is hell. There is nothing worse in the world than being able to hit everybody in the room but yourself, and you can’t fucking get high now. […] You’re sick. You’re more addicted to just getting that needle in than you are to even the drug. It’s crazy. But she got on fentanyl, and she’s smoking it and she stopped at the needle.*

#### Changing overdose risk

Bereavement and personal experience of fentanyl overdose was a prominent theme in conversations with participants. Eric, a man in his 50s (SM; SF), experienced deteriorated vein health after years of injecting heroin, but recalled losing friends to overdose as a compelling motivator in his transition to smoking:

*…there’s everybody that was using needles with fentanyl, they were dying, and, like, that’s just–I don’t want to die. I’m just a junkie. I’m just trying to circle–looking for help. I’m–I’m–I have–I have hope one day that I won’t be. But, yeah, so I don’t–I didn’t shoot that stuff because I just–I seen how it was–it was taking bodies. So many people dying from shooting fentanyl.*

Oliver, a transgender woman in their 30s (SM; SF; IM; IF), who never smoked heroin and was a “big advocate for IV use”, initially resisted injecting fentanyl after losing several friends to overdose and observing that those who injected were more vulnerable to overdose than those who smoked:

*I seen a lot of my friends overdosing. And the less overdoses I seen was with my friends that smoked it than my friends that were IV users.*

While our participants typically identified smoking as a safer alternative to injecting, a few strongly believed that smoking was still quite risky. Gena, a person in their 40s (SM; SF), emphasized the rapid impact of smoke inhalation contributing to respiratory depression.

*Interviewer: So why this overdosing from fentanyl? Is it–is smoking safer?*

*Gena: No, I wouldn’t say smoking is safer. Smoking fentanyl is a faster way to get high from it because it definitely goes straight to the brain […] and it could definitely slow down your breathing rate very fast without you knowing if you’re not paying attention because it puts you in a sleep state. And when you [are in a] sleep state […] you’re kind of relaxed, and if you’re too relaxed, what could happen? You’ll stop breathing.*

### Generating knowledge and techniques for smoking fentanyl

#### Cultural foundation for smoking

While fentanyl smoking is a recent development, several participants recalled experiences smoking other substances—cannabis, heroin and methamphetamine—as the knowledge base for their current smoking practices. Caroline, a woman in her 40s (SM; SF), used aluminum foil to smoke fentanyl as she had previously done with heroin:

*Interviewer: And how did you learn to smoke? Did someone teach you or?*

*Caroline: How to smoke fentanyl?*

*Interviewer: Yeah.*

*Caroline: Well, you smoke it just like black [tar heroin].*

**Fig 2:**
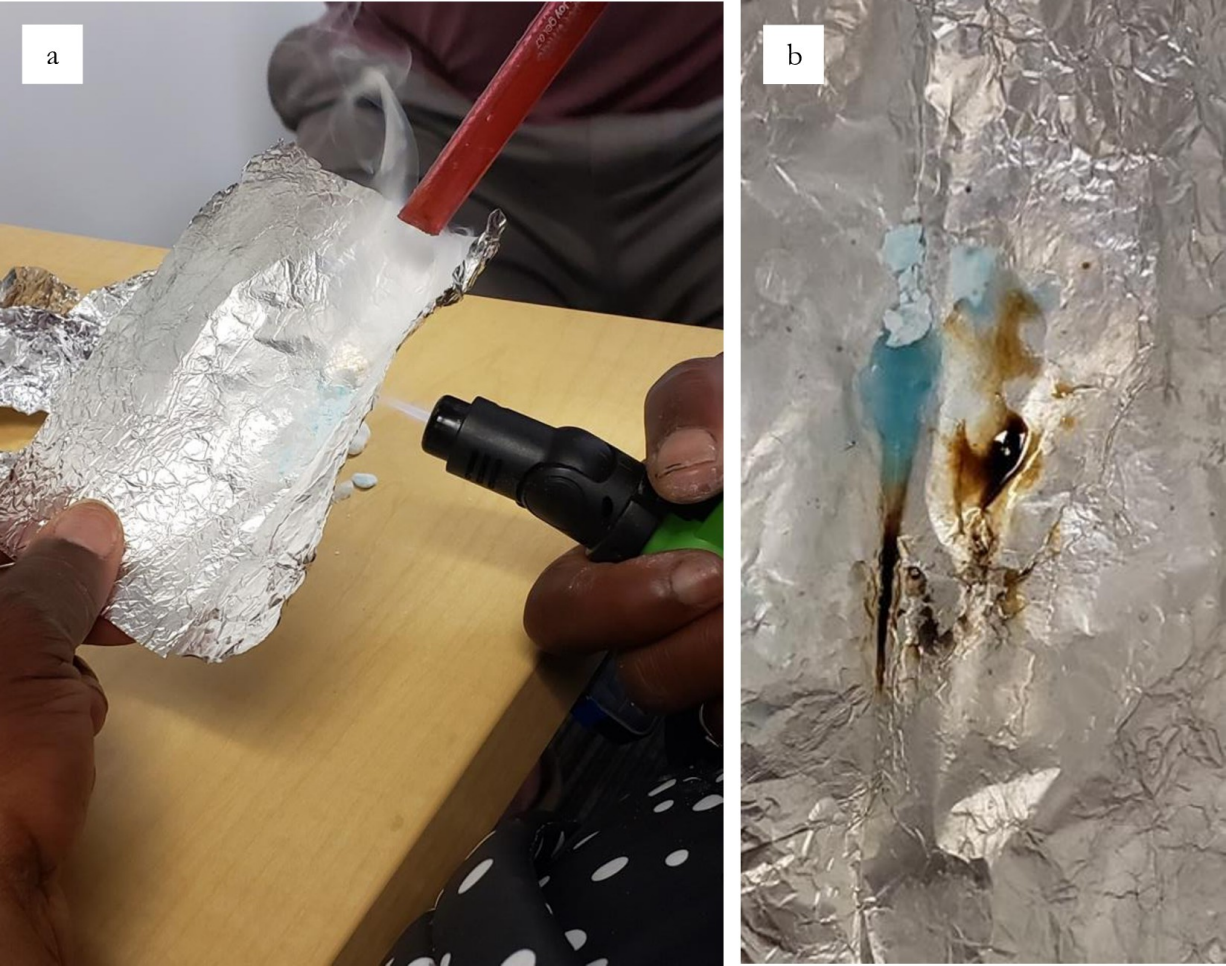
**a.** Demonstration of fentanyl consumption using the foil technique, **b.** Fentanyl salt in solid, liquid and burnt forms.

**Fig 3:**
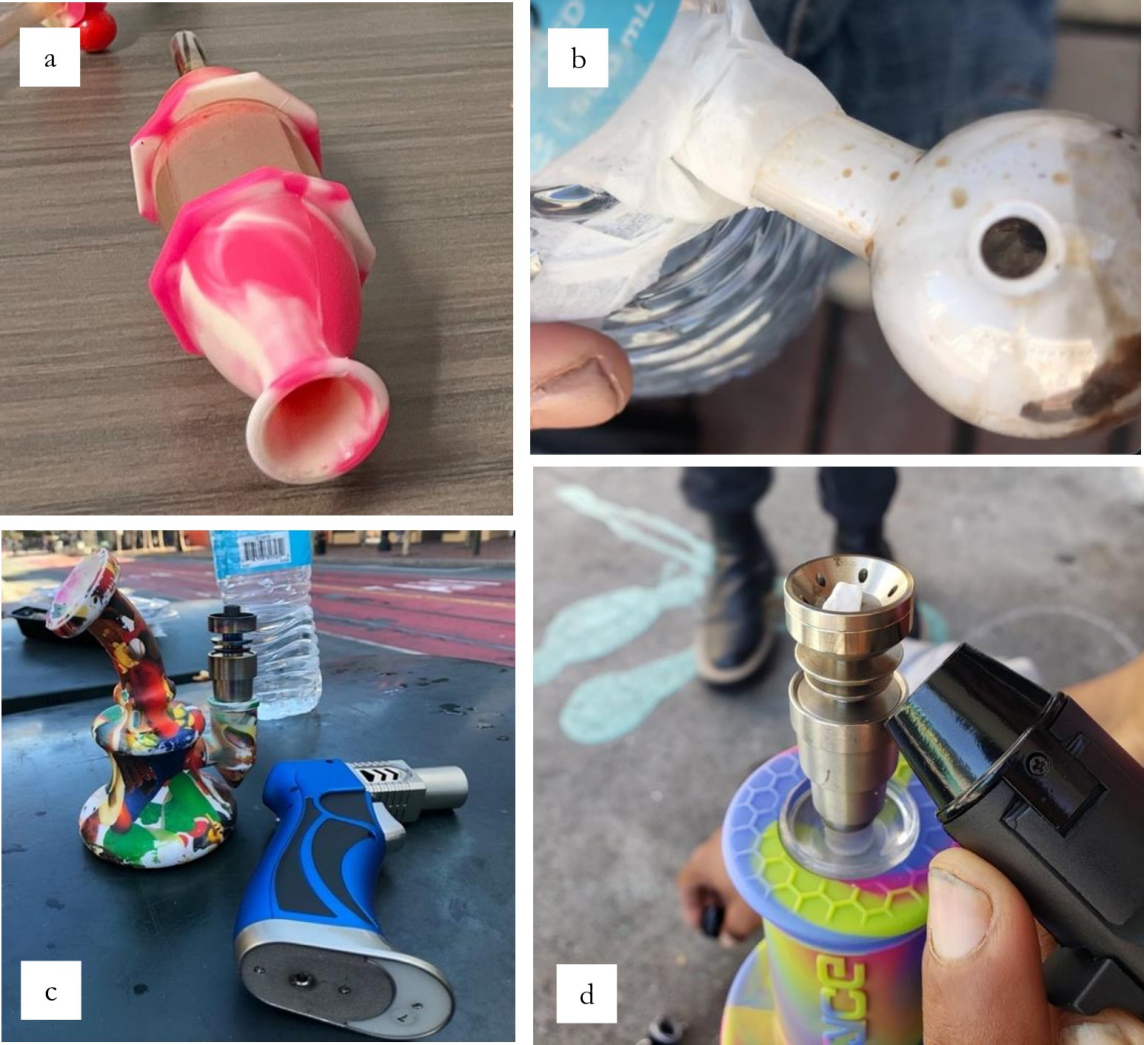
**a.** dabbing device, **b.** homemade bong, **c, d.** elaborate silicon bongs and torches.

Fentanyl can be smoked on foil or with a glass pipe (e.g. the ‘hammer’ pipe in Fig 1), and while foil was the most commonly used device observed among our sample, there were widely divergent views on the attractions and drawbacks of each smoking method. With the foil technique participants placed a chunk of fentanyl atop a small rectangular piece of foil and applied heat to the underside which produced a vapor for inhalation through a straw-like device, commonly referred to as a ‘tooter’ (Fig 2). Clay justified his preference for foil over a glass medium—usually a ‘bubble’ pipe (Fig 3A and 3B), commonly used for methamphetamine (‘speed’, ‘meth’ or ‘crystal’)—by noting that glass heated to too high a temperature, causing the smoke to be too hot to inhale:

*Normally, you just get a piece of foil and then just break off a piece of fentanyl on it. Then you find something to roll up like a dollar bill or a piece of paper or a straw if you have it or whatever, ignite it, and smoke it […]. Pretty straightforward process, really. Or you could use a piece of glass, but glass heats it up too much and then it becomes almost like null and void by the end of it. The smoke’s really hot, so you can’t even hardly inhale it, and then it just doesn’t affect the same.*

Despite majority of the group using foil to smoke fentanyl, several participants expressed health concerns from inhaling the vapors of heated foil. Russell both disliked the taste from smoking on foil and felt that it was “terrible for your brain”, while Kaila, a person in their 40s (SM; SF; IM; IF), shared their fears that foil vapors cause “Alzheimer’s and all that stuff.” Winston, a man in his 20s (SF; IM; IF), summarized a material trade-off that people must make, preferring to avoid the chemicals associated with foil but explaining that glass caused the fentanyl to burn (Fig 2B).

*Interviewer 1: Do you prefer foil over the glass pipe?*

*Winston: With fentanyl, yeah, because in the glass pipe you can’t–the heat doesn’t disperse as much, so it stays hot for a longer period of time, which causes it to burn quicker. It turns brown faster, which makes it taste bad.*

‘Dabbing’—a process commonly associated with the consumption of cannabis concentrates, e.g. hash, wax, shatter—also proved to be a popular technique among our group. The process of dabbing entailed intensively heating the tip of a ceramic, glass or metal tube-shaped device termed a ‘nail’ and touching the heated end to the drug, producing vapors for inhalation through the cool end of the tube (Fig 3A). The team observed some participants using this dabbing process with modest tools, while others had more intricate equipment. Tera shared her dabbing technique using a simple metal pipe with a silicone handle, expressing that less drug is wasted in the process:

*Tera: When I’m not broke and poor, I usually dab mine. So, it’s a metal tip. It’s a silicone handle with a metal tip. And you heat it with the torch until it’s like orange, and you touch the fetty rock, and it makes it smoke.*

*Interviewer: What is that? What is that called?*

*Tera: Dabbing. It’s dabbing wax like with weed. Yeah, you just touch it. Because it’s hot, and it makes it smoke, and you waste less of your dope that way.*

Like dabbing equipment, bongs were also popular and ranged widely in their design from large and complex to smaller, more transportable, silicon variations that cost roughly $20. These were used by several in our sample and other participants shared with us entirely homemade variations (Fig 3B–3D). Eden, a woman in her 40s (SM), showed our team how to create a bong using a simple plastic water bottle and glass bubble (Fig 3B). Using a bong produced a much smoother hit that participants found desirable, however, it also allowed much stronger hits—which, while suitable for some, caused overdose concern for others.

#### Regulating potency

While experience smoking other drugs often formed the cultural foundation for fentanyl smoking, the outsized overdose risk of fentanyl compared to previously smoked substances required participants to generate methods for regulating potency. Some believed the equipment played a significant role in moderating the strength of the hit, with glass being the least potent, followed by foil, then bongs and dabbing devices.

*Equipment*. Despite being the most common material we observed, some participants believed foil to pose an increased overdose risk compared to glass. Dylan, a man in his 30s (SM; SF), recounted foil’s involvement in most overdoses experienced and witnessed:

*The two times that I OD’d that I can recount, both times, I was hitting the foil…That’s why I shied away from foils for a long time, and I was like, you know, I respect the foil, is what I used to say. You know, I would only smoke out of glass. I feel, with glass, you get better hits, but I think that there’s something in the aluminum that, combined with, you know, heat and the fentanyl, that, I mean, most times, when… […] I’ve seen people OD it’s from hitting the foil.*

Kenny, a man in his 30s (SM; SF), was also wary of the risks of foil and advised on a stepwise progression through smoking equipment, in accordance with experience:

*Interviewer 2: […] what would you have a new person that’s first using fentanyl use?*

*Kenny: […] if they ask me, they say, what you think the best thing to use off safely, I would say a bubble. You know, you want to step it up? A hammer. A hammer is a step above a bubble because a hammer, it’s guaranteed for–for the most part it’s guaranteed glass. And, so, it’s a hard glass, though, and that hammer is, woo, that hammer do one. Woo. If you know what you’re doing, it’s all right. Then you graduate to the foil. […] Like, foil is big dog, but that dab, when you see how many people dab as opposed to how many people use the bubble, the hammer, and the foil, you going to see why the dab’s the heavyweight champion.*

Like Kenny, Lana echoed that dabbing produced the strongest hit, even beyond injecting:

**[…] It’s just way stronger*. *It takes it to another level where you don’t really need to slam* [inject]. *You don’t need to slam no more*. *You get higher dabbing than you’re going–you can’t get higher*. *You just can’t get higher*.*

Ethnographic observation supported the belief that bongs and dabbers provided stronger hits over alternative methods. Members of the research team observed two people using a bong and foil in sequential smoking sequences. Both video-recorded smoking sequences showed entire chunks of fentanyl evaporate upon touching the hotplate of a bong, allowing for only a single inhalation. Conversely, equally sized chunks of fentanyl using the foil technique lasted several hits over an elongated timeframe. For people with high tolerance, like Russell, who consumed two to three grams of fentanyl per day, bongs and dabbers proved to be the only smoking method that produced a sufficient high, potentially mimicking the bolus effect of injection:

**When I smoke out of the skillet* [the hot plate of a bong], *I smoke through two grams in*, *like*, *couple hits it seems like*.*

*Heat*. As well as varying the smoking equipment, some people also used heat strength to regulate potency, voicing that higher heat created more smoke, resulting in a stronger hit. Kaila voiced that she used a weaker flamed Bic lighter because “if I put a torch on the bubble, it smokes too much, and I might get too big of a hit, too.” Kenny noted the overdose risk from using a more powerful torch:

*I can’t smoke off a torch. Like, if I got my foil, I can’t smoke off a torch. It’s too hot for me. I hear it, boom, boop, somebody Narcan [Kenny]! Boop. […] Like, I’m gone.*

### Consumption together and protecting others

#### Fentanyl residue

**Fig 4:**
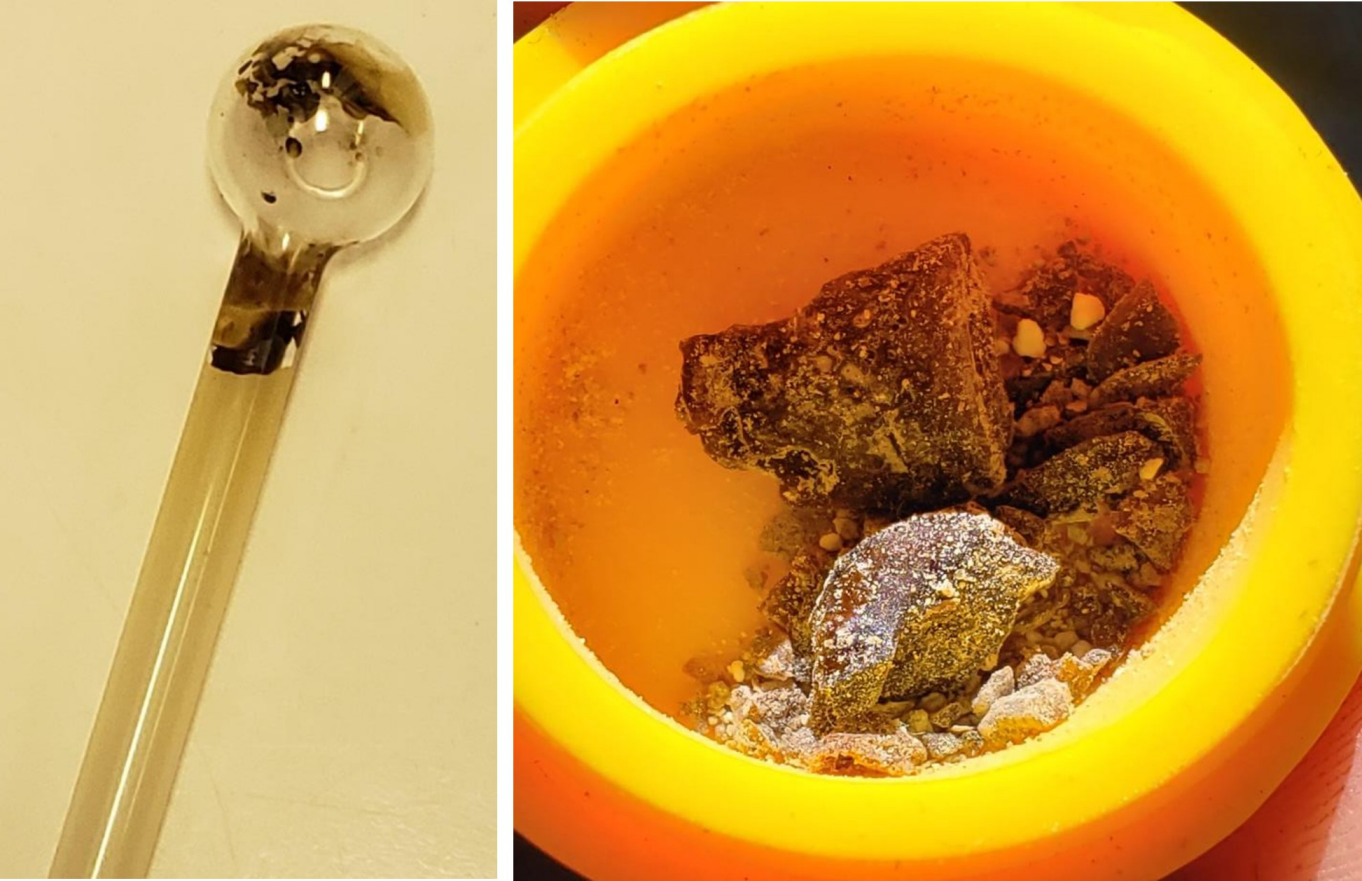
Resin buildup on equipment and collection in containers.

Through exploring the culture of smoking in San Francisco, the phenomenon of fentanyl residue or ‘resin’ became increasingly apparent. As people smoked fentanyl, a waxy, brown residue remained on the smoking equipment, accumulating over time, and representing a historical record of drugs consumed on that device (Fig 4). For Chris, this drug combination produced a “different type of high” that he found appealing, so much so that it dictated his ‘tooter’ preferences:

*I prefer using metal or glass tubes though because the resin will get, will accumulate, and then you can push it out or melt it out, and smoke that, and it’ll be—it’ll collect over time. All the different types of whatever you’ve been smoking, resin will collect. So you’ll get a nice mix of like 12 different types of drugs…*

Some people found this resin so desirable that they collected it in containers (Fig 4), however, not everyone felt this way. Several participants would not smoke the residue themselves but disclosed giving it away freely. For instance, Russell called himself “picky” and didn’t “like the way it taste[d]*”*, but agreed that the resin was potent and would donate it to others:

*Interviewer: So what–what do you do with the residue from–*

*Russell: […] most of the time it–it will save up. It will get all, like, real dark brown and there will be, like, little pieces of fentanyl in there, and I take the nail off, you know, and take, like, a paper clip or something and I’ll scrape it out of there, but I don’t–I don’t smoke it. I give it away or give it to other people. But that stuff is really, like, really strong […]*

Chris highlighted a cause for concern regarding shared resin, noting uncertainty of both what drugs were smoked to build up that residue and the tolerance of the recipient.

*Interviewer: Now wouldn’t also this idea of resin […] wouldn’t it kinda also be a little dangerous then? Like you know, what if somebody got your pipe…*

*Chris: Yeah and they cleaned all my resin [out of my bong] and they weren’t used to smoking ‘clean’ and I had a lot of ‘clean’ resin in there for instance, yeah they could OD if their tolerance isn’t high enough.*

#### Sharing equipment, drugs and overdose risk

In addition to resin sharing, interviews and observations revealed that several participants frequently shared their smoking equipment. While much of this appeared to be by choice, a staff member attending to the consumption space added that they frequently ran out of specific equipment—most notably pipes—which promoted sharing among the group. Conversely, Melvin, a man in his 40s (SM), attributed increased availability of smoking supplies from the service as contributing to reduced sharing of supplies.

*Interviewer: And do people share bubbles pretty often?*

*Melvin: They still do a little here and there. And they used to [use] a lot more. I used to get a lot more people, “Can I use your bubble? Can I fill your bubble, use your bubble, do you have a bubble?” Now that they actually give them out more, it’s a lot less. And then, you can always be like, “Dude, you don’t need my bubble. Because they’re giving them out right there, up the street.”*

Not all sharing was intentional, some was incidental. Team members observed people unknowingly drop their own supplies (e.g. paper straw tooters) and pick up someone else’s as their own. When asked explicitly about sharing equipment, the predominant concerns related to hygiene and communicable infections. Gena called sharing pipes “risky”, clarifying her unease as the result of “people putting their lips, saliva, bacteria in your or on your straws”. Oliver expressed a willingness to share their pipe with people “as long as they ain’t got no mono or anything like that…” While not mentioned by all, some participants—like Shawn, a nonbinary individual in their 40s (SM; SF; IM; IF)—related concerns around sharing to the recent COVID-19 pandemic.

*Interviewer: Is there any sort of ethic out there around sharing, and borrowing, and lending your bubbles?*

*Shawn: Me, especially since COVID, I’m not smoking after anybody. But yeah, other people are still, like passing a pipe between each other.*

Participants discussed sharing not only equipment, but also drugs. During a video-recorded smoking sequence, one participant inhaled a hit and then proceeded to share the foil with people sharing the sidewalk space, adding that he would have also shared his ‘tooter’ but they already had their own. On occasion, participants even offered drugs to members of the research team, although of course these were refused. In the Tenderloin area, this sense of community was commonplace, however participants perceived other neighborhoods as being hostile towards both fentanyl itself and people who use it. These experiences in combination with the concentration of services and a large street economy seemed to foster a more social atmosphere that was conducive to a community around smoking in the downtown neighborhoods.

For some, the question of sharing equipment and drugs evoked a sense of responsibility for the overdose risk of others. Lana indicated that she has a much higher opioid tolerance than others, and tailored the type of equipment she used and would share depending on the social scene in which she found herself. Her concern was partly motivated by protecting others but also the avoidance of unwelcome attention from law enforcement arising from overdoses.

*Interviewer 1: When do you use one [smoking device] rather than the other?*

*Lana: It depends on how I feel or how strong my fetty is or who I’m smoking with…*

*[…]*

*Interviewer 2: Is this somebody that’s opioid-naive?*

*Lana: Somebody that just doesn’t have a high tolerance. But a lot of people will be like, “I can outsmoke,” and fentanyl’s not a fucking game or a joke. You’ll fucking die, and I don’t want that on my record. If you’ve never smoked before, I’m not smoking with you. And if you don’t have a high tolerance, I’m not going to pull out something stupid for you to smoke on. You’re not going to smoke on my bong or my dabbers. It’s not going to happen.*

Clay also conveyed concern for the overdose risk of his peers and distinguished between different individuals he was willing to share with:

*Interviewer 1: Do you share a lot?*

*Clay: I try not to, honestly. […] I tend to not share, either, because I don’t want to be the person to give somebody something and for them to fall out or whatever and it be my fault, as far as that goes. Now, if somebody’s terribly sick and it’s clear that they use drugs constantly or I know them really well, I would probably help them out in that situation […]*

However, his concern for protecting others was not universal. Clay continued that *“*most people don’t think about that for two seconds,” and are impressed that he has. Jamie, a woman in her 30s (SM; SF; IM; IF) was hesitant to share for hygiene purposes, only upon further questioning did she emphasize that overdose risk to others was also a significant worry, explaining a recent occurrence where she shared her foil:

*Interviewer: What about in terms of, like, tolerance of the person. Are you ever thinking like, oh, they don’t use fentanyl, or they do, or they use meth, or …*

*Jamie: Oh, yes […] that’s a big thing. Like, if someone is coming up and I don’t know what their tolerance is or what their habit is, like, it’s definitely mentioned, like, prior, hey, this is ‘clean’, it’s not like normal stuff, you can’t just–you know, if you haven’t been smoking for a while or whatever, like. Because, you know, I’ve talked to several people who, like, let someone hit their foil or whatever and then they OD’d, which actually just happened to me recently. And I didn’t–like, it was, like, a random situation…Well, and the guy was saying that he was oh so sick, and he hadn’t smoked in, like, forever, and he needed to smoke […]so, I said, okay, here you go, this is really good stuff. He was right next to where the people that deal the good stuff were, so I kind of thought, okay, he knows what’s up, he’s here where they sell clean, like, I shouldn’t have to say anything, he said he was sick. Well, I let him hit my foil and, you know, I’m kind of doing my own thing. Like, and I don’t hear him say anything else or do–you know, I’m like, hey, how’d that go, did you like that? And I look over and he’s, like, crumpled into, like, a little ball and […] I ended up having to, like, resuscitate him, basically. Like, I gave him a Narcan, I, you know, did some, like, CPR on him and kind of, like, brought him back.*

For Josh, the social nature of sharing smoking equipment—along with assumptions about the purpose of each type of equipment—resulted in him accidentally using fentanyl after assuming that a glass bubble contained methamphetamine, which is typically smoked in a glass pipe:

*I tried [fentanyl] once on–on a–on accident […] It was just–it was just really, like, just really, like, powerful. […] I was, like, no wonder people can–can die just from smoking this stuff; you know? Because, I mean […] it was in a–a speed pipe; you know? And I was under the impression that it was speed, and I–and I tried it, and then I, yeah, it was–yeah, it was–it was pretty strong.*

Early during fieldwork, we observed an interaction in which a random person attempted to borrow a glass pipe from a participant, who vehemently refused. The participant explained that the pipe had been used for fentanyl and did not want to share it with someone who only used methamphetamine. Chris explained this general concern for the overdose risk of others as perhaps a ‘unique’ aspect of San Francisco:

*[…] there is a lot of trust on word of mouth, like, with meth user to meth user or whatever, and San Francisco is a pretty unique place. Most people don’t have malevolent intent, so most people aren’t trying to, like, unconsciously kill somebody, or, you know, unwittingly OD them with fentanyl. So, it’s a communal type of thing, like, ’cause that hyper-cautiousness isn’t just with meth users. A good deal of fentanyl users don’t want to hand a pipe that they know or would suspect had fentanyl in it.*

### Evaluating risk

#### Consumption dose and frequency

Both those injecting and smoking described consuming multiple grams of fentanyl daily. Aiden, a man in his 50s (IM; IF), estimated two grams daily, while Russell indicated two to three grams. Some participants used less, for example Cedric, a man in his 40s (SM; SF), estimated that a gram would last three to four days, while Randall, a man in his 30s (SM; SF; IM; IF), used roughly a gram each day. However, some used considerably more. While filming a smoking sequence with another participant, Gena—while smoking fentanyl herself—called out to members of the team that we might want to speak with her as someone “who really knows what is going on”. While possibly an exaggeration, she said “Fentanyl I would smoke all night like three grams back to back and be fine. Like, I’ve had six grams of fentanyl before talking to you guys…”

In addition to smoking large volumes, it was also common for people to report smoking frequently. Caroline, a woman in her 40s (SM; SF), estimated 1–2 hits per hour, saying “I’m not proud of it, but I smoke continuously throughout the day”. Karl smoked hourly, sharing that with smoking, “you need it more, and more, and more…” but that “it’s more of a mental thing…” and that physical sensations of withdrawal would not commence until the end of the day. Along those lines, several participants reported smoking whenever they are “bored”, with Russell saying he will “smoke just to smoke”. Oliver emphasized how the commensal nature of smoking influenced its frequency, saying “It’s became more of a pastime than a treatment for a pain. It’s like a social thing”. Andie, a woman in her 30s (SM; SF; IM), fears that this constant nature of smoking is contributing to overdoses because people are simply “not paying attention to how much [fentanyl] they’re smoking”.

#### Burns and fires

The heat required of the smoking process introduced a new hazard—burns and fires—reflecting the high risk of combining sedative drug effects with naked flames. Russell had several visible burns from touching hot parts of smoking equipment, saying he “nods out” and burns himself “quite a bit actually”, but also shared experiences of severe fires resulting from knocking over his torch in the locked ‘on’ position. In addition to hot equipment and direct flames, the fentanyl itself caused severe burns that Dylan revealed:

*I mean, these are fentanyl burns. These are burns from the residue from fentanyl, and this one is gnarly. I’m just covered, I mean, you know, with burns. Just it slides out, and it’s hot oil, boom, boom!*

## Discussion

An organic smoking culture has arisen in San Francisco that is historically significant, i.e., the first known example of a synthetic opioid being widely smoked in the US, and shows elements of both innovation and adaptation. The possibility of smoking fentanyl, whether for an intense bolus-like high or a milder effect, is offering a new potential for non-injected opioid use. Our findings are consistent with “diffusion of innovation” theory, i.e., a social learning process by which members of a group accept new ideas either through opinion leaders, or due to quick understanding of the benefits over costs/risks [31]. Innovation has historically been considered a force in changing drug trends and modalities, e.g., increased licit and illicit use of the hypodermic syringe, initially to inject morphine, following its invention in the 19^th^ century [14]. More recently, a hypothesis using diffusion of innovation theory suggested earlier uptake of smoked, aka ‘crack’, cocaine use among individuals already using ‘hard drugs’ [32]. Cultural knowledge transmission (e.g., doses, equipment, heating, wind protection) foments the population-level innovation of smoking and includes adaptation of elements from other drug using cultures, e.g., cannabis and methamphetamine, which have long traditions of smoking. While diffusion of innovation theory may be relevant, its application to public health is not without limitations. One possible critique is that it focuses too greatly on individual choices. Further research taking into account the broader context and social dynamics should be considered [33].

Our findings highlight an important novel risk factor for overdose: sharing of the bioactive residue or resin left in the smoking equipment. This could be seen as analogous to the risk of shared injection paraphernalia and HIV transmission [34–36]. Smoked fentanyl and methamphetamine residues look similar and the equipment used often overlaps. While many persons use both methamphetamine and fentanyl there is a sizable population who solely use methamphetamine. Our data highlight this as a new overdose risk as the residue is quite frequently shared, traded or stolen, with potential mismatch between the potency of the residual drug and the tolerance of the recipient. Harm reduction-based and culturally attuned education campaigns need to be rapidly advanced to address this new risk.

An important question is broached but not satisfactorily answered by this research: how does smoking impact overdose risk? Many participants held the notion that smoking fentanyl posed a lower risk of overdose than injecting it. Smoking heroin is considered more protective of overdose, compared to injection [37], due to potentially slower intake and lower blood concentrations, i.e., decreased bolus effect. Recent data showing a self-reported reduction in non-fatal overdoses among people who smoke fentanyl compared to people who inject fentanyl suggest this may also be true to some extent for the more potent opioid, fentanyl [38]. Much of the smoking consumption we witnessed involved smaller doses with high frequency, compared with those seen in our previous research with heroin/fentanyl via injection [25, 39, 40]. However, the opposite potential is also true: we witnessed folks possibly mimicking injection-like boluses by smoking large amounts with single bong hits. San Francisco has seen high levels of overdose since 2019, with 2020 surpassing 700 overdose deaths. Unfortunately, preliminary data reveal that 2023 has overtaken 2020 as the deadliest year on record, with 806 overdose deaths [41]. In addition, we observed extraordinary daily doses of fentanyl being consumed. For example, we regularly heard or observed persons consuming a gram, or more, of street fentanyl per day. Assuming 5% purity by weight (a strong possibility since the local spectrometry machines can routinely pick up fentanyl and their lower limit of detection is ~5%), this infers an approximate dosage of 50 mg of pure fentanyl/day–an extraordinary amount (but not including losses to the environment). The impressively low cost and high availability of IMF likely enables this level of consumption. More research is needed on dose, frequency, total daily consumption of smoked fentanyl, plasma blood levels, and comparing routes of use.

As well as promoting camaraderie among people smoking fentanyl, the shared smoking culture that we saw in parts of SF could both decrease [42] and increase risk. A number of participants described the precautions they took to prevent their opioid naïve peers from using their smoking equipment and overdosing on the bioactive fentanyl residues. Sharing saliva borne pathogens when passing pipes, bongs or tooters was a prominent concern among those we spoke with. There were also hints that larger quantities than intended were sometimes consumed during social participation than alone. If Andie’s observation that people are “not paying attention to how much they’re smoking” is correct, this coupled with the low cost and high availability of IMF, sociability and other factors are leading to exceedingly high consumption rates. This highlights the need for data that can inform harm reduction education that is understanding of and responsive to the perceptions of PWUO.

In previous work we have highlighted some of the harm reduction techniques used by participants to (potentially) ameliorate overdose risk from injecting fentanyl-adulterated heroin: snorting, aka “tooting,” a bit before injecting, judging the potency by “taste,” and utilizing tester shots, i.e., smaller, or more dilute amounts, before committing to the full dose [25]. Similarly, fentanyl smokers also have notions of regulating potency, and thus possibly altering overdose risk, through lower heat (ordinary lighter vs torch) and different equipment (bubble vs hammer vs foil vs dabber), and different dosing techniques.

Recommendations to reduce the risk of overdose from fentanyl smoking–go slower, reduce dose/increase frequency, keep track of consumption, don’t share equipment/residue until you confirm the tolerance of the recipient–are potentially viable interventions and deserve further exploration. Many carry and report using naloxone to reverse overdoses; interventions to promote naloxone carry [43] may have greater impact in this commensal environment. In addition, we need further investigation into why some PWUO continue to inject, with an aim to develop interventions to enhance transitions to smoking.

The increasing spread of xylazine adulterating fentanyl supplies is also of particular concern [44]. One study participant who took his supply of ‘clean’ fentanyl for drug testing found that it was adulterated with xylazine, the first such result in San Francisco. The implications of smoked xylazine/fentanyl combinations are unknown.

While the potential for respiratory complications is an unknown, smoking over injecting fentanyl has possible benefits to individuals and society: reduced burden from HIV/HCV transmission, as well as from injection-related bacterial infections, e.g., in the tissues of the skin, bone, heart, etc. [45]. HIV diagnoses have been rising nationally for White adults, in the transmission category of injection drug use, from 2013 to 2021 [46] and several local HIV outbreaks have been documented in the eastern and mid-western US [47, 48]. It is quite possible that if smoking becomes a norm among fentanyl users across the US, then HIV incidence in this population will drop. However, open hostility to harm reduction services is also evident in the US, with successful calls by some politicians in 2022 to prevent pipes from being added to safer smoking kits in federal funding streams for harm reduction organizations [49]. Ensuring adequate safer smoking supplies is paramount given the social environment and elevated risk of sharing resin. This concern is compounded locally by the closure of the Tenderloin Center, a crucial harm reduction service in San Francisco, in late 2022.

The results presented are from qualitative research using purposive, non-random sampling and as such are limited in generalizability. Other groups, places and contexts will likely yield different findings. Qualitative research methods offer greater depth in cultural and contextual understandings and can generate hypotheses for quantitative testing. Social desirability may have introduced some bias, however triangulation of data between interviews and observations aids in improving validity.

### Summary

Our work represents a “deep dive” into the nascent culture of fentanyl smoking. This innovation is likely to advance across the country as some of the risks of injection, e.g., vein loss and infections, are ameliorated, while drug effects are maintained or enhanced. Stigma is reduced while commensality is enhanced. Overdose risk is a wildcard: the use and sharing of drug residue is a highlighted new risk. Ethnographic research, such as presented here, can help in designing much-needed culturally appropriate and acceptable interventions, especially harm reduction-based programming.
